# Stages of changes for fruit and vegetable intake and their relation to the nutritional status of undergraduate students

**DOI:** 10.1590/S1679-45082014AO2926

**Published:** 2014

**Authors:** Lígia Cardoso dos Reis, Ingrid Chaves Correia, Edna Shibuya Mizutani

**Affiliations:** 1 Universidade Nove de Julho, São Paulo, SP, Brazil

**Keywords:** Fruit, Vegetables, Feeding behavior, Food habits, Students

## Abstract

**Objective:**

To assess the nutritional and dietetic profile of freshman Nutrition undergraduate students, and its association with stages of changes (Transtheoretical Model) for fruit and vegetable intake.

**Methods:**

Demographic (age and gender), anthropometric (body mass index and waist circumference) and nutritional (pattern of fruit and vegetable intake) data were obtained. The Transtheoretical Model was used to identify the stages of change for fruit and vegetable intake. Food consumption was assessed with a questionnaire developed by the Ministry of Health. The significance level considered for all statistical tests was 0.05 (p<0.05).

**Results:**

From 433 eligible students, anthropometric measurements were taken from 219 (50.6%), and 299 (69%) underwent food intake evaluation. The sample included undergraduate students with a low frequency of adequate fruit and vegetables intake (29.8%), being the majority (64.9%) of them classified as at the preparation stage to increase the intake of these food groups. Prevalence of adequate fruit and vegetables intake was higher among students at the action/maintenance stages (83.3%) compared to those at the precontemplation/contemplation (18.3%) and at the preparation stages (32.0%). Students at the preparation stage presented the highest medians for body mass index (p=0.004) and waist circumference (p=0.039) compared to those at the precontemplation/contemplation stages. There was no association between fruit and vegetables intake and the presence of overweight or abdominal obesity (p=0.373).

**Conclusion:**

This instrument is effective to predict the food intake and, even among aware individuals and ready to change their food behavior, the prevalence of nutritional risk is high.

## RESUMO

**Objetivo:**

Avaliar o perfil nutricional e dietético de universitários ingressantes no curso de Nutrição, e associá-lo aos estágios de mudança do comportamento (Modelo Transteórico) para consumo de frutas e hortaliças.

**Métodos:**

Foram coletados dados demográficos (idade e gênero), antropométricos (índice de massa corporal e circunferência da cintura) e nutricionais (perfil de consumo de frutas e hortaliças). Foi aplicado o Modelo Transteórico para investigar o estágio de mudança comportamental para consumo de frutas e hortaliças. O consumo alimentar foi avaliado com instrumento empregado pelo Ministério da Saúde. Para todos os testes estatísticos foi adotado nível de significância 0,05 (p<0,05).

**Resultados:**

Dos 433 alunos elegíveis, 219 (50,6%) participaram da antropometria e 299 (69%) da avaliação do consumo alimentar. A amostra foi constituída por universitários com baixa frequência de consumo adequado de frutas e hortaliças (29,8%), sendo a maioria classificada na preparação (64,9%) para aumentar a ingestão desses alimentos. A frequência de consumo adequado de frutas e hortaliças entre universitários na ação/manutenção (83,3%) foi superior à identificada entre aqueles na pré-contemplação/contemplação (18,3%) e na preparação (32,0%). Os alunos na preparação apresentaram maiores medianas de índice de massa corporal (p=0,004) e circunferência da cintura (p=0,039) em relação aos pré-contempladores/contempladores. Não foi identificada associação entre consumo de frutas e hortaliças e excesso de peso ou obesidade abdominal (p=0,373).

**Conclusão:**

O instrumento utilizado é eficaz em predizer o consumo alimentar e, mesmo entre indivíduos mais esclarecidos e preparados para a mudança comportamental, a prevalência de risco nutricional é elevada.

## INTRODUCTION

The last Household Budget Survey (POF, acronym in Portuguese for *Pesquisa de Orçamentos Familiares*), performed between 2008 and 2009^(1)^ by the Brazilian Institute of Geography and Statistics (IBGE), showed that overweight has been increasing in the adult population since the 1970s, and currently may be found in about half of the Brazilians. These changes in the nutritional epidemiological profile of the population characterize the process called nutritional transition. According to the World Health Organization,^(2)^ this phenomenon includes qualitative and quantitative diet changes of the population, such as an increase in energy density of meals and less consumption of fruit and vegetables. The Risk and Protective Factors Surveillance System for Chronic Non-Communicable Diseases through Telephone Interviews (VIGITEL)^(3)^ identified that only 18.2% of the Brazilian population consumes five or more servings of fruit and vegetables daily.

Considering the complexity of the diet interventions to improve eating habits, often dietitians face low compliance in patients with the guidance given.^(4)^ The methods to evaluate food intake and dietary guides currently adopted do not take into consideration the cognitive and emotional dimensions of eating behavior. In this way, a holistic approach is vital in order to encourage individuals to adopt eating patterns that promote health.^(5)^ It is crucial, for example, to understand why people do not consume adequate quantities of fruit and how the intake of this food group can be increased.^(6)^

One of the instruments developed to clarify the manner in which people change their behavior is called the Transtheoretical Model of Behavior Change (TTM). TTM was initially developed by Prochaska and DiClemente to describe the process of behavior changes in addiction. This model describes how people modify a problematic behavior or acquire a positive behavior, considering that behavioral change involves processes that evolve from a series of stages.^(7)^ Identification of a patient's modification stage can help healthcare professionals in individual counseling.^(8)^ TTM aids in the preparatory evaluation of patients to alter their lifestyles, thus directing more effective action strategies.^(8)^ Prior identification of the various stages of behavior modification can contribute towards outlining more assertive nutritional education programs, which encourage people to promote concrete and long-lasting changes in their diets.^(5)^ Likewise, knowledge of frequency, distribution, and determinants of fruit and vegetable consumption in the country is fundamental for the proposition of strategies that stimulate the population to consume these foods.^(9)^

## OBJECTIVE

To evaluate the nutritional and diet profile of undergraduate students, and associate it with behavior change stages regarding the consumption of fruit and vegetables.

## METHODS

The sample of this cross-sectional study was composed by universe of undergraduate students entering the Nutrition course in the first semester of 2012, at *Universidade Nove de Julho* in the city of São Paulo.

Data collection was carried out from May to November, 2012, in two phases: in the first, anthropometric data (weight, height, and waist circumference – WC) were collected, and in the second, the sociodemographic (age and gender) and nutritional (fruit and vegetable consumption, and eating behavior change stages) data were collected.

Gathering anthropometric data was performed at the Nutrition outpatients clinic and at the nutritional evaluation laboratories of the university. In order to verify the anthropometric measurements, the techniques proposed by the Ministry of Health were adopted.^(10)^ Weight was obtained using Filizola® electronic platform scales, with 100g precision and 150kg capacity. The participant stood, with no shoes or excessive clothing, in the center of the scales to equally balance weight on the feet. Height was measured with the patient in an erect position, with relaxed arms, feet together united at the heels, calves, gluteus muscles, shoulders, and head next to the SECA® anthropometer with 0.1cm precision fixed to the vertical surface. The head remained in the Frankfort horizontal position for verification. WC analysis was made with the patient standing, with the measuring tape positioned over the midpoint between the last rib arch and the iliac crest, with pressure sufficient for it to adhere to the body. Reading was made at the moment of expiration.

Weight and height measurement allowed the calculation of the body mass index (BMI) to then classify the nutritional diagnosis according to the parameters suggested by the WHO. Adults were diagnosed as being low weight (BMI<18.5kg/m^2)^, eutrophic (BMI≥18.5kg/ m^2^ and <25kg/m^2)^, or overweight (BMI≥25kg/m^2)^,^(11)^ while for the adolescents, the BMI curves for age were used (Z score).^(12)^

WC was used to identify the presence of risk of metabolic complications associated with abdominal obesity. As per WHO,^(13)^ these risks are increased when the WC of adults is >94cm for men and >80cm for women. The WC of adolescents was evaluated according to parameters established by McDowell et al.^(14)^ using the over 75 percentile to diagnose abdominal obesity, as suggested by Savva et al.^(15)^

In the second phase of the study, those evaluated responded to a questionnaire drawn up by the Ministry of Health^(16)^ for analysis of regular intake of fruit and vegetables, and an instrument developed by Greene et al.,^(17)^ in order to identify behavior factors related to the regular intake of fruit and vegetables. There are five stages of change described by Prochaska e DiClemente:^(18)^ pre-contemplation, when the individual does not have the intention of modifying his/her behavior in the next 6 months; contemplation, when the individual considers changing behavior in the next 6 months; preparation, when individuals actively plan a change for the next month; action, when the individuals have already made the change and have shown commitment to the new behavior for less than 6 months; and lastly, maintenance, when the commitment to the new behavior is maintained for more than 6 months.

Three indicators of fruit and vegetable consumption were created, based on the classification proposed by Jaime et al.^(9)^ for “regular consumption” and “recommended consumption” of these food groups. From these criteria, the category of “unsatisfactory consumption” was defined. The variables created were: regular consumption of fruit and vegetables (consumption equal to or more than 5 days in the week of these two food groups); adequate consumption of fruit and vegetables (consumption of these foods five or more times a day); and unsatisfactory consumption of fruit and vegetables (consumption of these foods less than 5 days a week).

The students that incorrectly filled out the self-applied evaluation questionnaires on the behavior change stages and on the consumption of fruits and vegetables were excluded from the analysis.

To characterize the study population, a descriptive analysis of the variables was made using absolute and relative frequencies, measurements of central tendency (means and medians), and dispersion (standard deviations - SD, and minimum and maximum values). For the associations among the categorical variables, the χ^2^ test was used. Relations between categorical and quantitative variables, without normal distribution, were assessed by Mann-Whitney's non-parametrical tests for two categories, and Kruskal-Wallis's for three categories, followed by Tukey's post-hoc test, when p<0.05. For all statistical tests, the statistical level of significance adopted was <0.05 (p<0.05). Analyses were performed using the Statistical Package for the Social Sciences (SPSS), version17.

The present study was approved by the Research Ethics Committee of the *Universidade Nove de Julho* (Protocol no. 437856 and Official Opinion no. 112.223) as to the respective Informed Consent Form, in order to initiate data collection.

## RESULTS

During the data collection period for the present study, 433 students were enrolled in the first semester of the Nutrition course. However, only 219 agreed to participate in the collection of anthropometric data, 303 of them present on the day the questionnaires were applied. Two refusals to answer the questionnaires were recorded and two instruments were incorrectly filled out. Since it was not possible to establish posterior contact with these students, the final sample was composed of 299 interviewees (69% of the study population), 219 of them with anthropometric data collected (50.6% of the study population). Considering it was a study conducted with university freshmen, the sample comprised adolescents aged 17 to19 years and adults aged 20 to 55 years. Table 1 demonstrates the characteristics of the population studied. It was noted that the sample was composed primarily of women and individuals with inadequate consumption of fruit and vegetables. Despite this food consumption profile, most of those evaluated were classified as being in the stage of preparation to increase the intake of these food groups.

**Table 1:** Demographic, nutritional, and behavioral characteristics of the study population

Variable		n (%)	Mean	SD
Age (years)			23.5	6.7
	17-19	108 (36.1)		
	≥20	191 (63.9)		
	Total	299 (100.0)		
Gender				
	Female	273 (91.3)		
	Male	26 (8.7)		
	Total	299 (100)		
BMI			23.2	4.5
	Malnutrition	17 (7.8)		
	Eutrophic	142 (64.8)		
	Overweight	60 (27.4)		
	Total	219* (100.0)		
	BMI* - adolescents		21.9	3.6
	BMI* - adults		24.2	4.8
Cardiovascular risk (WC)				
	High	54 (24.7)		
	Low	165 (75.3)		
	Total	219* (100.0)		
	WC (cm)* – adolescents		73.4	10.1
	WC (cm)* - adults		77.6	10.6
Consumption of fruit and vegetables				
	Adequate	89 (29.8)		
	Regular	158 (52.8)		
	Unsatisfactory	52 (17.4)		
	Total	299 (100.0)		
Stages of behavior changes				
	Pre-contemplation	55 (18.4)		
	Contemplation	38 (12.7)		
	Preparation	194 (64.9)		
	Action	4 (1.3)		
	Maintenance	8 (2.7)		
	Total	299 (100)		

SD: standard deviation; BMI: body mass index; WC: waist circumference.

* In 80 individuals, anthropometric data were not collected.

A significant association was observed between age range and nutritional diagnosis by the BMI analysis (p<0.05). Among those with overweight, 19.1% were adolescents and 33.1%, adults. In the same way, WC measuring enabled identification of a greater proportion of adults with an elevated risk for cardiovascular disease (34.6%) relative to the adolescents (10.1%), with p<0.001.

Table 2 illustrates the relation between the profile of fruit and vegetable consumption of the population studied and the stages of behavior changes, grouped into pre-contemplation/contemplation (contemplation), preparation, and action/maintenance. It was noted that most of the undergraduate students in the action/ maintenance stages had an adequate consumption of fruit and vegetables. Additionally, no cases were identified among these individuals on unsatisfactory consumption of these food groups (χ^2^=26.013; p<0.001).

**Table 2:** Relation between the pattern of consumption of fruit and vegetables with the stages of eating behavior changes of the study population

Food consumption profile	Contemplation* n (%)	Preparation n (%)	Action/maintenance n (%)
Adequate	17 (18.3)	62 (32.0)	10 (83.3)
Regular	52 (55.9)	104 (53.6)	2 (16.7)
Unsatisfactory	24 (25.8)	28 (14.4)	0 (0.0)
Total	93 (100.0)	194 (100.0)	12 (100.0)

χ^2^ test=26.013; p<0.001.

* The “contemplation” category refers to the pre-contemplation and contemplation stages.

Table 3 shows the differences in medians of BMI and WC among the eating behavior changes stages, grouped as pre-contemplation/contemplation (contemplation), preparation, and action/maintenance. A statistically significant difference was identified between the stages of contemplation and preparation, both for BMI and for WC. Table 3 demonstrates that the undergraduate students in the preparation stage showed greater BMI (p=0.004) and WC (p=0.039) medians as compares to those in the contemplation stage.

**Table 3:** Relation between eating behavior change stages with the body mass index and waist circumference of the study population

Stages	BMI	p value	WC (cm)	p value
Contemplation*	21.3 (4.4)	0.004**	73.0 (12.1)	0.039**
Preparation	23.2 (5.6)	0.002***	75.0 (14.0)	0.045***
Action/maintenance	21.0 (5.8)		72.5 (23.1)	

BMI: body mass index; WC: waist circumference.

* The “contemplation” category refers to the pre-contemplation and contemplation stages;

** Kruskal Wallis test;

*** post-hoc test.

Value expressed by median and interquartile intervals.

Although the BMI mean and median of the undergraduate students with an adequate consumption of fruit and vegetables (mean=24.1; SD=5.5; median=22.3) were greater than the means and medians of those with unsatisfactory intake (mean=22.0; SD=3.8; median=21.0), no statistically significant difference was observed between the BMI means and medians in the fruit and vegetable consumption groups (p=0.132). The undergraduate students with a regular consumption of these foods had a mean BMI=23.2 (SD=4.0) and a median of 22.5.

Likewise, the WC mean and median among the individuals with appropriate consumption of fruit and vegetables (mean=77.6cm; SD=12.2; median=752cm) were superior to the means and medians of the undergraduate students with unsatisfactory (mean=73.3cm; SD=7.8; median=73.0cm) and regular (mean=75.8cm; SD=10.4; median=74.0cm) consumption of these foods. Nevertheless, no statistically significant difference was observed between the means and medians in WC of the fruit and vegetable consumption groups (p=0.319).

Also tested (χ^2)^ was the association between the categories of fruit and vegetable consumption (adequate, regular, and unsatisfactory) and the classes of nutritional diagnosis by the BMI (malnutrition, eutrophic, and overweight) and by the WC (with cardiovascular risk and low risk). However, no statistically significant difference was observed (p=0.334 and p=0.284, respectively) between these variables. Also not identified was a statistically significant association between the lower frequency of fruit and vegetable consumption and the presence of nutritional risk (overweight or abdominal obesity), with p=0.373.

As to the consumption of fruit, it was noted that mean daily intake of glasses of natural juice was 0.7, with a SD=0.9. The means of days in the week and of times a day in which the population evaluated consumed fruits were 3.8 (SD=2.2) and 1.5 (SD=1.2), respectively. Although it was not investigated if the consumption values differed statistically, the mean weekly consumption of raw salad (3.8; SD=2.2) was higher than that of cooked vegetables (3.2; SD=2.1). Those evaluated reported that they ingested, on average, at least one vegetable on 4.4 (SD=2.2) days a week. There was a greater frequency of raw salad consumption at only one main meal (67.4%) relative to those who have the habit of consuming this preparation at two meals (28.2%), or of not inserting it into the diet (4.4%). The same pattern of behavior was observed for the intake of cooked vegetables: 11.1% reported not eating them, 58.7% ate them at only one primary meal, and 30.2% at the two main meals of the day. However, these differences were not tested as to statistical difference.

## DISCUSSION

The present study identified a high proportion of individuals with inadequate consumption of fruit and vegetables, although it was made clear that most of those evaluated are in the preparation stage to modify this behavior.

According to Jaime et al.,^(9)^ the studies that verify the profile of fruit and vegetable consumption, such as the VIGITEL system,^(19)^ may enable evaluating the impact of government programs directed at promoting the consumption of these groups of foods in the country. For Jorge et al.,^(20)^ analysis of the consumption of these foods may enable the identification of the determinants of their inclusion in the diet.

Despite the increased availability of foods in the household, the mean consumption of fruit and vegetables of the Brazilian population is still half of that recommended by the Eating Guide for the Brazilian Population.^(21)^ The latest data from the VIGITEL System^(19)^ indicated that only 20.2% of those interviewed reported ingesting five daily servings of these foods. Although the present study does not quantify the servings of fruit and vegetables, it may be affirmed that a low level of consumption of these foods was identified among undergraduate students. Inadequate patterns of fruit and vegetable consumption were evidenced in studies carried out with adolescents and adults.^(22–24)^

The identification of sociodemographic, cultural, and cognitive/emotional determinants of daily eating can facilitate the compliance of patients with the nutritional treatment.^(25)^

Intervention strategies to increase the intake of fruit and vegetables based on TTM proved to be effective in some studies.^(17,22)^ The identification of the stages of behavior changes allows the formulation and application of nutritional strategies that encourage individuals to adopt adequate eating practices, thus increasing the impact of actions that promote health^(5)^.

According to Johnson et al.,^(26)^ individualized interventions, based on behavior change stages, can lead patients in pre-action (pre-contemplation, contemplation, and preparation) to the stages of action/maintenance.

The findings of the present study suggest that the instrument proposed by Greene et al.^(17)^ is effective in predicting the profile of fruit and vegetable consumption, since individuals in the action and maintenance stages did not show unsatisfactory consumption of these foods.

The sample of the present study comprised mainly women, and a high prevalence of nutritional risk was identified (overweight, abdominal obesity, and inadequate consumption of fruit and vegetables) in the undergraduates evaluated. As expected, most of these Nutrition students are in the preparation stage to modify the frequency of fruit and vegetable consumption. Besides being undergraduate students of the healthcare field, it is important to point out that in this course, there is a predominance of women, which could also explain such findings. For Campos et al.,^(27)^ women can have their food choices influenced by more interest in nutrition and healthy foods. This influence might explain why they showed more favorable attitudes towards eating fruit and vegetables.^(22)^

Despite the WHO^(2)^ considering the regular consumption of fruit and vegetables a prevention factor against obesity, the findings of this study do not point to a relation between the presence of overweight and abdominal obesity with the pattern of consumption of these vegetables. However, in spite of the fact that these relations are not statistically significant, it was noted that the BMI and WC of the undergraduate students were greater among those who consumed these foods five times a day. Neutzling et al.^(23)^ stated that there is a possibility of bias of reverse causality in the association between fruit and vegetable intake and nutritional status, since the obese can modify their food consumption as a consequence of their nutritional state.

In the same way, in associating the stages of behavior change with the nutritional profile of the undergraduate students, it was possible to identify those in preparation who presented with higher BMI and WC means relative to the contemplative undergraduate students. A study conducted by Wee et al.^(28)^ to evaluate the factors associated with the more advanced strategies of behavior change in patients of Primary Healthcare demonstrated that the individuals who perceived weight as a risk to health showed a greater possibility of being in the preparation, action, and maintenance stages for weight loss, diet improvement, and exercise. The population of this present study, which already showed a higher incidence over the relations between eating, nutrition, and health since it was composed of undergraduate Nutrition students, could have been encouraged to initiate the process of change in consumption of fruit and vegetables (preparation) due to dissatisfaction with body weight.

Institutional data demonstrated that the family monthly income of 47% of the freshman in the Nutrition course of the university evaluated corresponded to up to two minimum wages. This social vulnerability has been considered an important barrier to increased consumption of fruit and vegetables.^(24)^ For Barreto et al.,^(29)^ some issues, such as income, frequency of meals outside of the home, and low compliance with healthy food at cafeterias at work and school hinder the adoption of appropriate eating habits. At the university evaluated in this study, the food court had mostly fast foods, snacks, and pizzas. The lunch options that included fruit and vegetables were scarce and had a greater cost in comparison to the other preparations mentioned.

Since the sample of the present study comprised adolescents and adult undergraduate students, the nutritional and diet profile observed is alarming and reflects a prognosis of chronic non-communicable diseases. The habitual intake of vegetables at only one main meal and of fruit only four times a week by the undergraduate students interviewed shows the inadequacy of the consumption of these food groups, as recommended by the Eating Guide.^(21)^

A study conducted by Silva & Petroski^(30)^ confirmed the tendency towards modification of lifestyle after entering college, contributing to the increased health risk of the students assessed. Consequently, actions that provide the creation of institutional environments that promote adequate and health eating, as recommended by the National Policy of Eating and Nutrition, are essential for the undergraduate students to have healthy behaviors, bearing in mind that eating habits acquired during undergraduate school may persist in the following years.^(24)^

This study has as limitations the heterogeneous sample, composed of men and women, from diverse age groups and socioeconomic brackets; the excessive loss of samples relative to the universe proposed, which can generate a profile of evaluated individuals which is not representative of the Nutrition course of the university; the absence of anthropometric data on many of those evaluated; the absence of sociodemographic data and reference to clinical history of the participants, which could be determinants of consumption of the foods addressed; and the instrument used to evaluate the adequacy of the consumption of fruit and vegetables, which does not allow the collection of data in servings or grams, as recommended in the Eating Guide^(21)^.

## CONCLUSION

The findings suggest that, even among more educated individuals in more advanced stages of behavior change, the prevalence of nutritional risk (overweight, abdominal obesity, and insufficient consumption of fruit and vegetables) is high. This evidence could have been determined by behavioral, social, economic, and environmental factors. The instrument used to evaluate the intention to modify fruit and vegetables consumption proved to be effective in predicting the consumption profile of these food groups in the population evaluated. Considering that this population is in the preparation stage to modify its eating behavior, it is vital to provide favorable environments for adopting healthier eating patterns. Therefore, a greater offer of fruit and vegetables in the college environment, with lower prices, is fundamental for the promotion of healthy eating, appropriate for the social and cultural aspects of undergraduate students.
